# Publishing FAIR Data: An Exemplar Methodology Utilizing PHI-Base

**DOI:** 10.3389/fpls.2016.00641

**Published:** 2016-05-12

**Authors:** Alejandro Rodríguez-Iglesias, Alejandro Rodríguez-González, Alistair G. Irvine, Ane Sesma, Martin Urban, Kim E. Hammond-Kosack, Mark D. Wilkinson

**Affiliations:** ^1^Center for Plant Biotechnology and Genomics, Universidad Politécnica de MadridMadrid, Spain; ^2^ETS de Ingenieros Informáticos, Universidad Politécnica de MadridMadrid, Spain; ^3^Department of Computational and Systems Biology, Rothamsted ResearchHarpenden, UK; ^4^Department of Plant Biology and Crop Science, Rothamsted ResearchHarpenden, UK

**Keywords:** FAIR data, Linked Data, Pathogen-Host Interactions, PHI-base, Semantic Web, Semantic PHI-base, SPARQL, data integration

## Abstract

Pathogen-Host interaction data is core to our understanding of disease processes and their molecular/genetic bases. Facile access to such core data is particularly important for the plant sciences, where individual genetic and phenotypic observations have the added complexity of being dispersed over a wide diversity of plant species vs. the relatively fewer host species of interest to biomedical researchers. Recently, an international initiative interested in scholarly data publishing proposed that all scientific data should be “FAIR”—Findable, Accessible, Interoperable, and Reusable. In this work, we describe the process of migrating a database of notable relevance to the plant sciences—the Pathogen-Host Interaction Database (PHI-base)—to a form that conforms to each of the FAIR Principles. We discuss the technical and architectural decisions, and the migration pathway, including observations of the difficulty and/or fidelity of each step. We examine how multiple FAIR principles can be addressed simultaneously through careful design decisions, including making data FAIR for both humans and machines with minimal duplication of effort. We note how FAIR data publishing involves more than data reformatting, requiring features beyond those exhibited by most life science Semantic Web or Linked Data resources. We explore the value-added by completing this FAIR data transformation, and then test the result through integrative questions that could not easily be asked over traditional Web-based data resources. Finally, we demonstrate the utility of providing explicit and reliable access to provenance information, which we argue enhances citation rates by encouraging and facilitating transparent scholarly reuse of these valuable data holdings.

## Introduction

### Traditional scholarly data publishing

As with all life science domains, research into plant diversity, biochemistry, development, and disease is becoming increasingly data-centric. There are more than 1800 entries in the latest compilation of life science databases undertaken by the Nucleic Acids Research journal (Galperin et al., 2015). Many of these are domain-specific, or special-purpose resources that are usually curated, and contain specific types of data (e.g., RNACentral for noncoding RNA sequences Bateman et al., 2011) or data relevant to a specific species (e.g., AraPort's collection of information about *Arabidopsis thaliana* Krishnakumar et al., 2015). However, in addition to these special-purpose repositories, there is also a proliferation of general-purpose repositories such as Dataverse (Crosas, 2011), FigShare^1^, Dryad^2^, Mendeley Data^3^, Zenodo^4^, DataHub^5^, DANS^6^, and EUDat (Lecarpentier et al., 2013) where researchers deposit a wide range of often specialist data and datatypes, at scales ranging from single figures or images, to large high-throughput datasets.

For most plant biologists, manually navigating these data resources to find, integrate, and analyse the data they require is exceedingly difficult. Not only is the data dispersed among many special- and general-purpose repositories, making it harder to locate, it is also often provided in formats that are difficult to integrate—both with the researcher's local datasets, and with data from other Web repositories and databases. Once located, the data may be difficult to interpret and/or be of questionable quality—in fact, a recent study into these problems in the area of ecology and evolution found that 64% of public data archives were “unusable” (Roche et al., 2015). All of this results in a great deal of error-prone, manual copy-and-paste by biologists, and increasingly requires the researcher to employ a bioinformatics specialist to write bespoke data retrieval, cleansing, and integration software.

While the problem of discoverability and reusability of publicly-archived data in general-purpose repositories will require both technical and social/behavioral changes—which will likely be driven by journals and/or funding agencies^7^ —advancing the state of special-purpose repositories is a more tangible near-term objective due to their more centralized and focused nature. Moreover, by and large, the same decisions must be made for both types of data resources, thus the knowledge gleaned from one activity can inform the other.

Locating and accessing data within special-purpose repositories should, in principle, be straightforward. These repositories generally have a curatorial staff concerned about quality-control, have organized the data into sensible structures, and have usually enriched the data with cross-references both internally between elements of their own data, and externally to remote datasets. Nevertheless, in practice it remains difficult to query, extract, and integrate the data, even from these highly curated repositories, particularly at the scale demanded by contemporary high-throughput research. Why is this so? What are the practices of data repositories, and the properties of their data holdings, that affect (positively or negatively) the usability of their valuable contents?

### Contemporary data publishing guidelines

The recently-published FAIR Data Principles^8^ (Wilkinson et al., 2016) are an attempt to answer these questions, and provide data producers and repository curators with a set of guideposts that lead to ever-increasing degrees of data discoverability and (re-)usability. FAIR is an acronym of Findable, Accessible, Interoperable, and Reusable. Briefly, the FAIR Principles suggest that every data element should have a globally-unique identifier, and that this identifier should be associated with contextual, searchable metadata (“Findable”); these identifiers should all resolve to data or metadata using an open, standard protocol (“Accessible”); the data and metadata should use a formal, broadly applicable representation language, and utilize open and widely-accepted domain-relevant vocabularies and ontologies (“Interoperable”); and finally, the data should be richly described with an abundance of cross-references, and with a clearly-defined mechanism for accessing provenance and license information (“Reusable”). Beyond the core principles, “FAIR-ness” also emphasizes that each of the principles should be equally applicable to both humans and machines. This is in recognition of the ever-increasing need for automation within large-scale data discovery, retrieval, integration, and analysis pipelines. Within that proposal were details regarding the kinds of qualities FAIR data would exhibit, and the integrative behaviors that would result. What was not defined by the FAIR Principles, however, was how this might be implemented, particularly over important existing data repositories.

While FAIR-ness speaks to more than just data representation and formats, and does not suggest any specific implementation or technology, in practice most FAIR transformations to date have utilized the principles of Linked Data (Bizer et al., 2009). Linked Data is both a design-philosophy, and a structured format. Linked Data was specifically conceived to work within the Web, and builds on the core technologies of the HTTP communications protocol, Uniform Resource Identifiers (URIs) for naming entities, concepts and relations, and Resource Description Framework (RDF) as a generic information model. The Linked Data principles require that all entities must be identified using URIs, that these URIs must resolve to information about that entity, and that information should include links to other entities also named by URIs. Thus, the Linked Data principles are more specific than, and span a subset of, the FAIR Data principles, where the latter additionally addresses “social” issues such as provenance, licensing, commitment-to-availability, and adherence to community-standards.

### From traditional to contemporary

Recently a series of BYOD (“bring your own data”) Workshops (Roos and Lopes, 2014), targeting specific research domains (for example, rare diseases^9^), have been established, where database owners are invited to work side-by-side with FAIR experts to learn about FAIR data representations, and to attempt to migrate portions of their data holdings toward FAIR-ness. The experience of the authors as trainers at these BYOD workshops, and other similar events, provided the impetus for this body of work.

Clearly, with nearly 2000 life science data resources in existence, bringing repository owners and FAIR Data experts together in the same room is not a solution that will scale, even within the single domain of life sciences. While FAIR Data workshops have resulted in the migration of certain limited data subsets toward FAIR-ness, and are thus a very promising start, few life science databases have been exposed in their entirety using the FAIR Principles. Unfortunately, there is scant information available to database owners about the decisions necessary to achieve the various facets of FAIR-ness, the process of implementing those decisions, the degree of difficulty of each implementation step, or the degree of benefit gained—by the data host, or their user-community. For example, certain aspects of FAIR are seemingly non-trivial to accomplish and might deter potential adopters; yet there are simple, but perhaps non-obvious solutions to achieving these goals. This general lack of structured guidance and cost/benefit evaluation hinders data repository owners, who will usually need to self-direct their FAIR implementation. As agencies and journals demand more rigorous attention to data reusability, we believe it is important and timely to provide such guidance, and explain the process and available resources, tips and tricks, that will lead to FAIR-ness for all valuable research data.

### An exemplar transformation

The Pathogen-Host Interaction Database (PHI-base Urban et al., 2015) is an important resource for plant sciences and molecular plant pathology researchers, and is one of the more than 1800 databases catalogued in the NAR Journal's database edition. While not limited to plants, it captures the data relevant to thousands of plant/pathogen interactions, the resulting phenotypes, and in many cases information about the molecular/genetic basis of pathogenicity.

The PHI-base interface is designed to support primarily manual exploration. Briefly, it consists of a Web based query form with both “simple” and “advanced” interfaces, allowing the user to do a universal keyword search, or select from a pre-populated set of filters that can narrow a search. Search results include hyperlinks to individual matching records. Each of these records contains a series of informational text fields such as gene name, submitter name, phenotype, and experimental evidence. Each record also includes (where possible) cross-reference hyperlinks to Entrez Gene, PubMed, QuickGO, and other third-party databases. The interface is clearly laid-out and all data elements are clearly labeled and straightforward to interpret by-eye. With respect to machine-accessibility, there is no application programming interface to the database, and the database itself is not available for direct query. Search results are provided in HTML rather than XHTML, and the HTML contains extensive formatting markup for human-readability, both of which would limit the ability of machines to “scrape” information from the results pages. Raw data is available for download after agreeing to a free-form description of terms and conditions of use, however the downloads cannot be accessed in any automated manner, and the download is all-or-nothing. Downloads are available in FASTA format, or in a bespoke XML format that lacks a formal Schema. The database XML file is approximately 7 MB.

For a variety of reasons, PHI-base represents an interesting target for wholesale FAIR transformation. First, it is relatively small with few data facets, and therefore its migration is a tractable problem for even a small investigative team with limited resources. It contains a variety of cross-references providing potential entry-points into domain-critical “big data” resources such as UniProt and PubMed, thus has the potential to demonstrate the added-value of dynamic, machine-guided data integration. And finally, it contains functional and phenotypic information of great importance to plant pathology researchers in academia, industry and government departments and other organizations, but which often requires integration with external data in order to be contextually understood or investigated. As such, a FAIR representation of PHI-base should provide immediate added value for its target community of researchers, and possibly for the curators of PHI-base itself.

On the basis of these considerations, we have undertaken to execute a FAIR transformation of the plant portion of PHI-base, and we refer to the resulting resource as “Semantic PHI-base.” While doing this transformation, we attempted to carefully document the decisions that were required and the rationale behind the decisions, the methodology of the transformation, and the particular “pain points” we encountered. As such, this study is primarily a methodology article; however, in the results section we describe the kinds of integrative and exploratory power that emerged as a result of executing this transformation.

Before we describe the methodology, it is important to note two considerations: First, although the FAIR Principles are designed to be modular, encouraging gradual, incremental steps of increasing FAIR-ness, the FAIR transformation described here is an attempt to be fully comprehensive, addressing every aspect of FAIR-ness, expressed or implied by the four principles. Second, with the exception of our semantic data model (for reasons described below), every implementation decision was made using the most simplistic or straightforward technological/architectural choice we could identify. We acknowledge that, in doing so, we paid no attention to aesthetics, nor (in some cases) even to common practices within the Linked Data community. Our primary goal was to transform an existing data resource into a form that exemplified, to the maximal extent practical and using minimal effort, the FAIR objectives.

## Materials and methods

### Starting data

The starting dataset was downloaded from the PHI-base database^10^, corresponding to version 3.7 of the data (May 9th 2015). The downloaded XML file contained 3369 gene records, 4792 interactions, 225 pathogens, 132 hosts, with 261 registered diseases and 1693 references.

### General workflow for data publishing

Though data publishing seldom follows a simple sequential workflow, we have followed the general guidelines for data publishing suggested by Villazon-Terrazas et al. (2012). They propose that data publishing proceeds through the phases of Specification, Modeling, Generation, Linking, Publication, and Exploitation, and give direction regarding the considerations at each step. We organize the methodological description below according to these phases.

### Specification phase

#### Selection of the data subset

In the specification phase, we must select what data is going to be published. We selected which PHI-base fields we wished to include in our transformation by visual inspection of the XML data model. We subjectively decided to restrict our export to the following fields (named by their XML tag that appears in the original data-dump): *PHI-base_accession, DB_Type, Accession, Associated_strain, Gene_name, Locus_ID, Pathogen_NCBI_Taxonomy_ID, Pathogen_species, Strain, Disease_name, Host_NCBI_Taxonomy_ID, Experimental_host, Function, Phenotype_of_mutant, Experimental_evidence, In_vitro_growth, AA_sequence, NT_sequence, GO_annotation, Literature_ID*. A description of these fields can be found online^11^.

The decision to limit our transformation to only the plant related portion of PHI-base was based solely on the limited domain-expertise of the authors; we did not feel sufficiently qualified to create, for example, controlled vocabularies in domains outside of our personal botanical/pathology experience. Exclusion of non-botanical records was therefore achieved by manual filtering on the basis of the authors' prior-knowledge of plant species names, and plant vs. animal pathogens.

### Modeling phase

Throughout this section, we will use italics to indicate terms (classes or properties) that are represented in the semantic model. For example, *Interaction* is the category of the model that describes the base entity of each PHI-base interaction record, and *is_manifested_as* is a property of *Interaction*.

#### Selection of an overarching semantic model

While FAIR Data does not demand a well-grounded semantic model, we wished to invest some effort into semantic modeling. Certain decisions at the model-level will enable us to more extensively use third-party vocabularies and ontologies downstream in the transformation process. This enhances both human and machine-readability and comprehensibility. By doing so, we rigorously address the Interoperability and Reusability principles of FAIR, which encourages re-using established vocabularies whenever possible (I), and using as many of these elements as possible (R).

As the base of our model, we selected the SemanticScience Integrated Ontology (SIO; Dumontier et al., 2014). SIO was specifically designed for the representation of scientific data, and is published under a Creative Commons license. It is therefore compatible with the “I” principle of FAIR, which requires that vocabularies be publicly available. The core semantic model of SIO breaks the world into Objects and Processes, each of which may have roles, capabilities, and/or qualities. It is, however, its approach to modeling observational data that is of most interest to us in the context of FAIR data transformation of PHI-base.

From a purely pragmatic perspective, the use of SIO forces us to model observations in a manner that is (a) easier for machines to explore, and (b) more amenable to capture of rich provenance information. For example, when using SIO, literal values (words, numbers, dates, etc.) are only allowed in the context of a single predicate—*has value*. As a result, observational data elements such as phenotypic descriptions cannot be attached to a Linked Data node by an arbitrary “*has description”* predicate. Rather, we are forced to model descriptions as *Description* objects in their own right, and these objects then have a SIO *has value* property leading to the textual data. This has two benefits. First, generic data-parsing software can be written to consume these data models, since the qualitative/quantitative data is always located in a predictable context (i.e., after the *has value* predicate). Second, since everything else must be modeled as an object, other useful metadata can be associated with these objects. For example, with the *Description* node just mentioned, we could add metadata properties related to authorship, revision date, citation information, etc. relevant to individual phenotypic descriptions. Thus, our selection of SIO makes it straightforward to adhere to the citation and provenance requirements of the FAIR Reusability (“R”) principle by providing numerous, high-granularity, yet predictable locations to carry this provenance data, and Findability (“F”) because these locations all have a unique and resolvable identifier, as described in Section What Elements Need to Be Identified, and How?.

#### What elements need to be identified, and how?

The FAIR and Linked Data Principles specify that all “entities” (i.e., things that are not textual or numerical values) must be named using a resolvable URI, and SIO provides a mechanism for defining a variety of types of entities, including both physical and conceptual entities. Clearly we must provide identifiers for the core informational components of PHI-base—interactions, hosts, pathogens, genes/alleles, observation protocols, literature citations, and phenotypes. We know, however, that the interactions between hosts, pathogens, and alleles are contextually-dependent. For example, the phenotype may only manifest under specific environmental or genetic conditions. Moreover, it is often the case in PHI-base data (as with many pathogenic observations) that the precise contextual details of the interaction are unknown, but may 1 day become known—for example, the specific allele sequence that leads to an altered phenotype. For this reason, we decided to include in our model a set of conceptual entities we refer to as “contexts.” There are contexts for interactions, for hosts, and for pathogens. An *Interaction Context* has properties related to the phenotypic description of the interaction in that context, including the disease name, and the scientific *Protocol* by which that interaction context was constructed (for example, a gene-knockout experiment). Within the scope of each *Interaction Context* are the *Host Context*, and the *Pathogen Context*. These two contextual nodes carry information that is specific to each of the interaction participants, such as their genotype (if known). As discussed earlier, the inclusion of these conceptual entities allows additional annotations to be associated with them, for example, the strain information or stock-center data associated with a particular pathogen.

Once the entities are enumerated we then must evaluate: first, which of these entities already has a stable FAIR identifier in some third-party database, vs. those entities which are unique to the dataset we are transforming; and second, whether these unique local entities are already represented by a local stable FAIR-compliant identifier. In the case of our context entities, the decision to “mint” new identifiers was clear, since these entities did not exist prior to our establishment of the Semantic PHI-base. In the case of other unique entities such as interactions, the decision was slightly more complex. There is a unique, pre-existing Uniform Resource Locator (URL) in PHI-base for each interaction; however, those URLs do not adhere to many of the FAIR principles, particularly with respect to resolving to machine-readable metadata. As such, we chose to also mint novel identifiers for these Interaction resources (note, however, that we utilize the pre-existing PHI-base identifiers at a later stage—see the “FAIR Accessor” section below).

In the case of entities that already have stable identifiers created by third-parties, we were faced with a different challenge. Good Linked Data practice, and the FAIR Principles, both direct us to reuse identifiers whenever possible. Unfortunately, several of the resources we considered critical with respect to our intended end-uses for Semantic PHI-base, such as cross-domain queries against major protein and genomic sequence databases, pre-date the establishment of these best practices, and/or the practices were not followed. As a result, there are often numerous, stable, yet synonymous third-party identifiers to choose-from when referring to common entities such as genes, alleles, and taxa. Choosing one or another dramatically simplifies federated query over resources that utilize that same identifier, but increases the complexity of federated query over resources that do not.

In response to the need to mediate this identifier proliferation, the Identifiers.org initiative (Laibe et al., 2014) emerged from the European Bioinformatics Institute. Identifiers.org minted a “canonical” set of identifiers, intended to represent all life science entities; however, these identifiers have an additional function to mediate discovery of synonymous identifiers. Resolving an Identifiers.org Uniform Resource Identifier (URI) provides a same-as mapping to equivalent identifiers in other datasets. Thus, in Semantic PHI-base, we decided to model all third-party concept identifiers using an appropriate Identifiers.org URI. Subsequent sections will reveal how those URIs can be used.

The final topic to address in this section is the structure of the URIs that must be minted to represent the local PHI-base concepts. Decisions about the structure of a URI (the “URI scheme”) are informed by three considerations: first, the ideas proposed by the REpresentational State Transfer (REST) software architecture (Fielding and Taylor, 2002); second, the need to design a simple resolver for all of our locally-defined URIs, in order to adhere to the FAIR principle of Accessibility (“A”); and third, the community consensus that stable identifiers should be “opaque,” i.e., that identifying concepts using, for example, sets of numbers, is preferred over identifying them by a name. We now explore these considerations in more detail, and how this led to our choice of the URI structure for Semantic PHI-base.

Although REST does not make any reference to URI structure, the REST principles discourage practices that expose the underlying interface. A straightforward and practical reason for this is that RESTful URIs are intended to have extreme longevity. However, the software that resolves an identifier today is unlikely to be the same software that resolves that identifier several decades from now. Therefore, identifiers that contain software-specific key/value parameters are unlikely to be stable over time. In our implementation, therefore, the scheme we select for our URIs will not utilize key/value parameters.

The second consideration is that the URI nevertheless must be resolved by software, and this software must be made aware of what identifier is being resolved. In common RESTful practice, the resolver software is identified by a segment within the path component of the URI. The software, when triggered, then examines the path information in the URI to determine the identity of the thing it is being asked to resolve. Thus, the structure of our URIs will include the name of the resolver software, followed by the unique identifier of the Semantic PHI-base entity.

The final consideration is that of identifier opacity. This is not only a recommended practice within the life science ontology community^12^, but an idea widely adopted by the data management community in general. In the context of the Web, having an opaque—e.g., numerical—identifier for a concept allows the concept, and/or the interpretation of that concept, to change over time, without triggering a desire to change its name. In addition, it encourages other best-practices in Linked Data, such as using labels on all entities, and including XML language tags on these labels such that all entities may be described multilingually. Therefore, the structure of our URIs will be opaque, and all entities will be properly labeled to achieve human-readability, as required by FAIR.

With these three considerations in-mind, our URI scheme follows this general structure:

http://linkeddata.systems/SemanticPHIBase/Resource/***type/ID***

where /SemanticPHIBase/ is used to partition this project from other similar projects; /Resource/ is the name of our resolver software (see the example code below); /type/ is a means of explicitly partitioning our identifier space into different domains of identifiers—examples of types would be /interaction/ or /hostcontext; and finally /ID is the identifier for the specific entity of that type, for example /HOSTCON_000123.

#### Selection of ontologies and vocabularies

In keeping with the intent of the FAIR Principles of Interoperability and Reusability, we reuse class names and property names from a wide variety of third-party ontologies. In most cases our prior awareness of the scope and content of each of these ontologies simplified our discovery and selection of the appropriate term; however, we utilized tools such as Protege (Knublauch et al., 2004), The BioPortal browser (Whetzel et al., 2011) and the ontology lookup service at EBI (Cote et al., 2010) to assist us in finding the most appropriate, and specific, class and property names.

The third-party ontologies we selected for the semantic model of PHI data include:

Relation Ontology *(RO)* (Mungall et al., 2015): Taken from OBO-Foundry. The predicates *has_participant* and its inverse *participates_in* have been used to model the relationships between individuals and the process that they participate in. In the SemanticPHIBase model, *has_participant* describes the relationship between an *Interaction* and its *Pathogen* and *Host*. The *depends_on* predicate was used to describe the relationship between an *Interaction Context* and its respective *Pathogen Context* and *Host Context*.

*Ontology for Biomedical Investigations (OBI)* (Brinkman et al., 2010): Taken from OBO-Foundry. The class *Organism* was used to categorize *Pathogen* and *Host* model nodes.

*EDAM* (Ison et al., 2013): EDAM contains numerous categorization branches for bioinformatics data and data-types, allowing us to very precisely characterize elements of the semantic model such as the specific type of id number contained in each cross-reference. The ontology terms selected include: *GO Concept ID, Protein Sequence, Protein Accession, Gene Name, NCBI Taxonomy ID, Locus ID, Nucleotide Sequence and PubMed Identifier*.

*Experimental Factor Ontology (EFO)* (Malone et al., 2010): The following categories were utilized from EFO: *Host, Pathogen, Disease*, and *Wild Type Genotype*.

*Sequence Ontology Feature Annotation (SOFA)* (Gene Ontology Consortium, 2006): We utilized the category *Gene* from SOFA to indicate the node-type of the genes, for which allelic variants are described within PHI-base.

*Schema.org* (Guha. R., 2011): We utilized the *CreativeWork* class and *citation* property.

Other properties and classes, and the overall root architecture of our data and semantic model, were adopted from the best-practices guidelines of SIO, as described above.

In cases where no appropriate third-party vocabulary contained a sufficiently descriptive term, we coined our own ontology term. Defining the URI structure for those terms was, however, unusually problematic. It is considered best-practice within the life sciences linked data community to utilize the Persistent Uniform Resource Locators (PURL)^13^ service to mint novel identifiers intended to exhibit long-term persistence. Unfortunately, at the time of our data transformation (and still at the time of writing) the PURL service had closed its administrative interface, thus precluding us from creating a namespace for our newly-minted identifiers. As such, we published our vocabulary on our own server, with the namespace prefix:

http://linkeddata.systems/ontologies/SemanticPHIBase#

Publication of vocabulary terms as globally-unique and resolvable URIs is a requirement of both Linked Data, and of FAIR (“F” and “A”), thus we ensured that the terms, and their definitions, could be retrieved from these URIs by HTTP GET.

#### The semantic model

**Figure 1:**
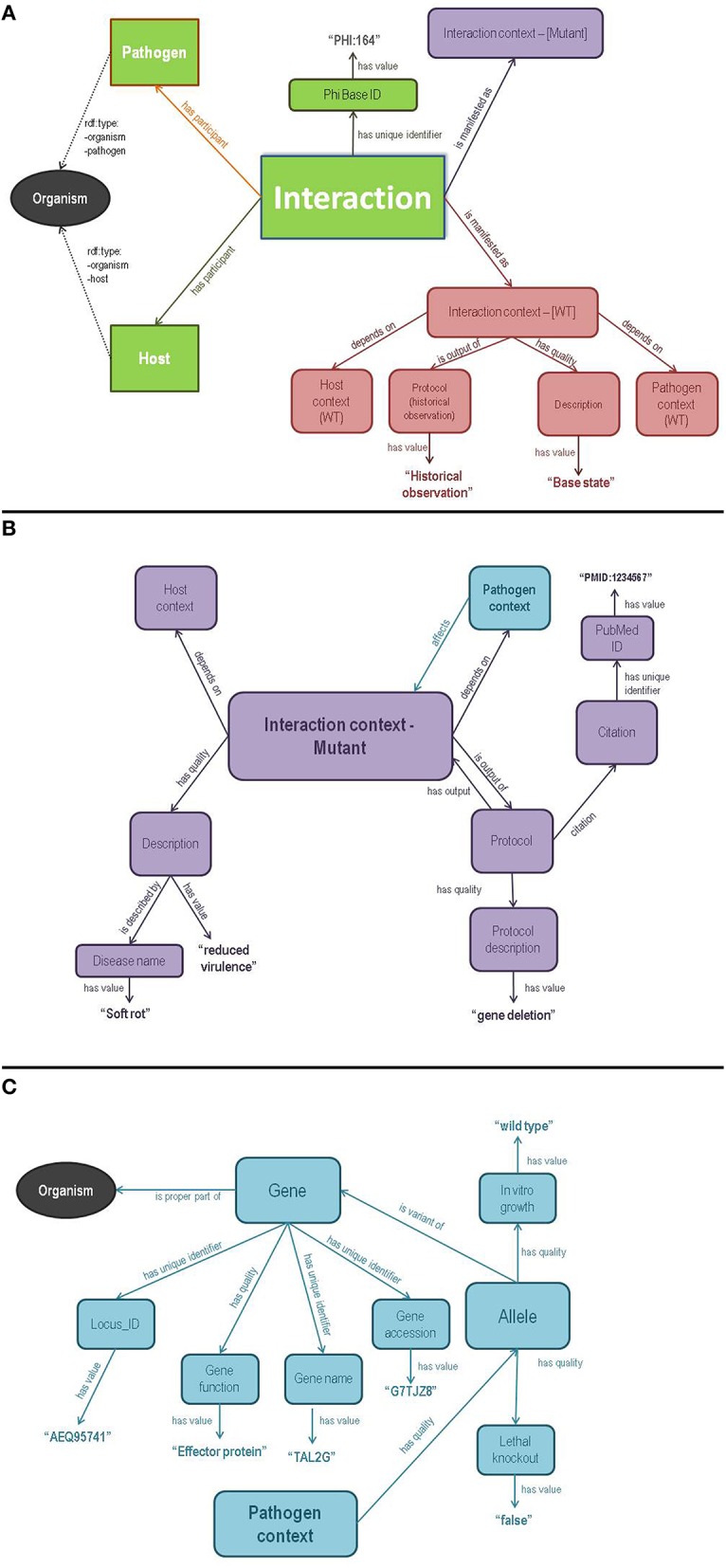
**An entity/relationship diagram showing a simplified model of the Semantic PHI base OWL ontology**. The three panels are color-coded such that entities shared between panels can be easily identified. **(A)** includes the core class—Interaction—and its primary participants of *Host* and *Pathogen* (green). The *Interaction* is manifested in both a wild type (red) and mutant (lavender) form of the *Interaction Context* OWL class. The elaboration of the wild type *Interaction Context* is also shown in **(A)** (red). **(B)** shows the overall structure of the mutant *Interaction Context* node (lavender), including the phenotypic *Description*, experimental*Protocol*, and *Citation* information. *Interaction Context* is connected to a *Host Context* and a *Pathogen Context*, of which only the *Pathogen Context* (blue) is populated with useful information. **(C)** shows the overall structure of the *Pathogen Context* class, which primarily connects to genetic information via an *Allele* class (representing the allele carried by that pathogen in that context). An *Allele* is linked to a *Gene*, which is extensively annotated into remote databases via *has unique identifier* properties, the values of which are, whenever possible, the Identifiers.org URL corresponding to that data-type in a remote database such as UniProt or EMBL. Finally, the *Gene* class links back to the *Organism* class (black), shared with **(A)**. Of importance is the fact that all “data”—that is, everything that is a value, rather than an entity, is connected to the model using the SIO property *has value*.

The semantic model behind the PHI-base Ontology was intended not only to reflect the knowledge contained in PHI-base, but to explicitly embody contextual and semantic information that is implicit in that database. Figure 1 shows the key elements behind the model, color-coded to show common elements between panels.

At the root of the model is an *Interaction* (Figure 1A), which has two participants (*has participant*): a *Pathogen* and a *Host*, both belonging to a general *Organism* class defined by two ontologies—OBI and SIO. *Organism* has properties including specific taxonomic information using Identifiers.org taxonomic identifiers. Each *Interaction* in Semantic PHI-base *is manifested as* one or more *Interaction Context* nodes. These nodes represent specific scenarios that influence the phenotypic outcome of that interaction, for example, whether the host and pathogen are in the wild-type or mutant state. *Interaction Context*, therefore, can be imagined as a container that *depends on* a unique combination of the three determinants of pathogenicity defined by the Disease Triangle (Agrios, 2007)—that is, the combination of *Host Context, Pathogen Context*, and environmental context that are required to achieve the manifestation of the pathogenic infection described by the *Interaction Context*. While PHI-base does not capture information about environmental determinants of pathogenicity, the *Interaction Context* node provides a sensible location to add an *Environmental Context* class in the future, should the information become available.

For every *Interaction* there are at least two *Interaction Context* nodes. One of these represents the “base state”—that is, the disease as it has been historically named and described, with a wild-type pathogen and a susceptible host (Figure 1A). This information is implicit within all PHI-base records, but our semantic model explicitly represents this important context. The second *Interaction Context* node (Figure 1B) models an atypical state captured by the PHI-base record—for example, the result of a mutant allele in the pathogen. This second *Interaction Context* includes details such as the *Protocol* by which the genetic modification was derived, the phenotypic outcome (*Description*), and *Citation* information. While our semantic model allows for any of the Host, Pathogen, or Environmental contexts to be informative within an *Interaction Context*, PHI-base primarily contains information about Pathogen variability. As such, only the *Pathogen Context* node is of-interest when modeling PHI-base variation data, and this is diagrammed in detail in Figure 1C.

The pathogen variation data in PHI-base is primarily molecular/genetic. Thus, *Pathogen Context* has a variety of SIO *has quality* relationships that capture this genetic variability in the *Allele* class. An *Allele* is described as a *variant of* a specific *Gene*, and a *Gene* is a *proper part of* a given *Organism*. A wide range of cross-references are attached to the Gene class, pointing-out to other resources such as UniProt and EMBL, using their Identifiers.org identifier. The *Gene* class is also the point of convergence between all *Interaction Contexts* that involve studies of the same gene; our model assumes that all alleles of that gene, in every *Interaction Context*, are distinct, given the lack of data to the contrary; however, all alleles of a gene share the same *Gene* node, which allows cross-query between *Interaction* nodes based on genetic information.

### Generation and linking phase

In contrast to Villazon-Terrazas et al. (2012), we consider these two phases to be inseparable, in that it is not possible to generate Linked Data that is not linked. Further, while they suggest using “same-as” relationships to create cross-references between one dataset and another, we propose that it is more appropriate (and more FAIR) to simply reuse the identifiers used by the third-party dataset. Finally, our utilization of the Identifiers.org mediator system obviates the need for any *post facto* linking phase, as we can dynamically create same-as linkages by querying the Identifiers.org resource (see Discussion section). As such, we address these two phases as a single step.

#### Data cleansing and harmonization

A deeper look at the PHI-base data composition revealed some format and syntax-related concerns, particularly with respect to inconsistent capitalization and punctuation of descriptive terms. This was most prevalent in the *In_vitro_growth* XML field. Solving this reduces term-redundancy, which enhances the ability to ensure comprehensive responses to queries over these fields. To achieve this, we followed an identity-mapping procedure: the same term, even if named differently, was assigned the same globally-unique URI (as required by FAIR's “F” Principle), and these were then published as a formal controlled vocabulary in OWL using the URI scheme for vocabulary terms defined above. The mapping files enabling this process were generated largely through a manual process of inspection.

A similar identity-mapping process was required due to the occasional use of organism common-names within the *Pathogen_species* and *Experimental_host* fields. In this case, the data was cleansed by manually mapping each common-name to its appropriate NCBI taxonomic name. The manually-generated mapping file was similarly used as input to an automated data cleansing script during data transformation. The NCBI taxonomic identifier number was also captured during this step, in order to create the canonical Identifiers.org taxon ID URI for these widely shared entities.

#### Data transformation

Data transformation was accomplished by a bespoke Java-based application. The application utilizes an XML DOM parser to consume the PHI-base XML. Conversion to RDF involved two phases, as follows:

- Data Harmonization and mapping
  ◦ *Scientific names*: Host and pathogen species, often identified by their common names in PHI-base, are mapped to their binomial scientific names using a manually-generated mapping file. From this, we further determine the taxon ID (at least to the level of the genus). While PHI-base records sometimes include strain information, this is not used at this step, since many of these strains will not have an associated taxonomic identifier in the NCBI taxonomy. The taxon ID is then used to generate an Identifiers.org URI which is used for the *Organism* model component.
  ◦ *Mutant phenotypes*: PHI-base phenotypic descriptions are not based on a controlled vocabulary, and thus have various inconsistencies in, for example, capitalization and punctuation (e.g.,: “Reduced virulence,” “reduced virulence”). We found it was not possible to automate the harmonization of these terms into a controlled vocabulary, and so this vocabulary was created manually by examining all descriptive fields, determining which ones were identical (semantically), and creating a controlled vocabulary class for each one. This became part of the SemanticPHIBase ontology, and these class URIs were used as the rdf:type of the *Description* model component.
  ◦ *Experimental evidence*: Similar to the situation with mutant phenotypes above, we undertook a manual harmonization of experimental evidence fields and created ontological classes for each of these in the SemanticPHIBase ontology. The resulting URIs were used as the rdf:type for the *Protocol* model component.
  ◦ *In vitro growth*: As above, the harmonization of *in vitro* growth data was accomplished manually.
  ◦ *Cross-reference prefixes*: PHI-base XML includes a “DbType” tag indicating the source database referred-to by the sequence identifier cross-reference. Where available, we use this tag to automatically generate an appropriate URI for that identifier, utilizing the Identifiers.org URI schema. For example, ENTREZ PROTEIN DbType maps to the http://identifiers.org/ena.embl/ URI prefix. These URIs were connected to the Gene model component using the *has_unique_identifier* property, and each was rdf:typed using the EDAM ontology, for example, http://edamontology.org/data_2907 “Protein accession.”
  ◦ *Disease URIs*: For each disease in PHI-base we manually looked-up the URI of the corresponding article in DBPedia, and where such a record existed, this was used as the identifier of the *Disease Name* class in our model. In other cases, we generated a local unique URI for that model entity. We made (limited) efforts to identify other cross-references, and these are connected to the model using rdfs:seeAlso properties.

- Conversion to RDF
  ◦ Each interaction record in the XML was consumed in its entirety, and was remodeled as RDF following the SemanticPHIBase ontological model using Apache Jena.
  ◦ Each model component (e.g., *Interaction, Interaction Context, Host Context*, etc.) was assigned a unique URI, which is simply an incremental alphanumerical identifier.
  ◦ Each model component was assigned a human-readable label (usually the model component type, its identifier, a textual value obtained from the PHI-base record, and the “en” XML language tag; for example “Host—Oryza sativa—HOST_01757”@en.
  ◦ Each model component was assigned an rdf:type, with the type being a shared ontological class from a third-party ontology and/or a class that is defined by our own SemanticPHIBase ontology. For example, the Oryza sativa host above is typed using four external ontology terms: EFO:EFO_0000532 (“host”); SIO:SIO_010415 (“host”); OBO:OBI_0100026 (“organism”); SIO:SIO_010000 (“organism”).

The version of the PHI-base XML we transformed contained 3341 interaction records, and this resulted in 827455 RDF triples.

With respect to FAIR, the transformation process includes several salient features:

1. Every entity is assigned a globally-unique, resolvable identifier.
2. It attempts to reuse existing identifiers whenever possible.
3. It attempts to reuse existing vocabularies and ontologies to annotate these entities.
4. It uses multiple, sometimes redundant, ontological references. Using the same concept from different ontologies broadens the range of agents that will understand the data, therefore the selection of ontologies and terms was made based on their usage within the community.
5. Newly-coined vocabulary terms are formally published in a FAIR manner.
6. Every component is labeled with human-readable text, including the language of that label.

#### Data linking

As described in the data transformation phase, we utilize Identifiers.org URIs to represent entities contained in third-party data resources. As such, the resulting transformed data is already “linked,” and no additional linking step is required. Third-party linkages included in Semantic PHI-base span literature (e.g., PubMed), DNA Sequence (e.g., EMBL), and Protein record (e.g., UniProt) cross-references. By re-using URIs created by third-parties within Semantic PHI-base, these cross-references become unambiguous and transparent, and our interoperability with those resources is enhanced.

### Publication phase

#### Selecting a triplestore

While the selection of a Triplestore technology is not a relevant consideration with respect to the FAIR Data Principles, we include here a brief discussion of our experience because we encountered significant pain-points at this stage of our project. While these obstacles will likely be short-lived—mostly the result of “bugs” in the triplestore software—we were nevertheless able to overcome them as a result of FAIR decisions we made earlier, thus providing an immediate demonstration of the utility of the FAIR data publishing approach.

Based on our previous experience with small-scale datasets, and the triplestore evaluations provided by Wu et al. (2014), we selected two triplestores to test—BlazeGraph^14^ (version 1.5.2) because of its ease-of-setup, and Virtuoso (Version 7.2.1.3214-pthreads) because it is widely used in the life sciences. Unfortunately, in both cases, we encountered problems with federated queries—a key requirement of our evaluation—however these had distinct causes. In the case of BlazeGraph, the message sent from the local BlazeGraph triplestore to the remote triplestore was not compliant with the SPARQL 1.1 specification, thus all federated queries failed (our bug report appears to have been entered into their software development workflow as of October, 2015^15^ so we anticipate that this problem will not persist). In the case of Virtuoso, while federated query itself worked, another aspect of SPARQL—the dynamic rewriting of URIs within a SPARQL query—was not working correctly in our installed version^16^. URI rewriting is commonly used in the life sciences to map between organizations that refer to the same entity in their respective triplestores using different identifiers. For example:

http://www.ebi.ac.uk/ena/data/view/Taxon:9606
http://purl.bioontology.org/ontology/NCBITAXON/9606
http://purl.uniprot.org/taxonomy/9606

all refer to the same concept—the taxonomy entry for *Homo sapiens*. Clearly, it is possible to map from one to another by simply changing the portion of the URL preceding the taxonomy number (9606), however this SPARQL feature was not functional in the version of Virtuoso used in this study.

Despite this barrier, we chose to continue with Virtuoso, and instead utilize the synonymous identifier-mapping capabilities of the FAIR Data resource Identifiers.org, as described in the Modeling Phase and Linking Phase sections above. Examples of using Identifiers.org to achieve federated queries are provided in the Discussion section.

#### Constructing a FAIR resolver for the minted URLs

As described above, in order to be FAIR, all locally-minted identifiers must resolve to useful data/metadata that can be read by both humans and machines. The URIs are coined within a namespace owned by our laboratory, therefore it is our responsibility to create that resolver software. While resolving a URI is not challenging *per se*, resolving it in a manner appropriate for both humans and machines poses more of a challenge, since RDF is not generally considered a human-readable format.

There are a variety of approaches and solutions to this challenge, with varying degrees of complexity. On the Web today, this is most commonly achieved through HTTP content-negotiation; software requests data from a resolver in a machine-readable format (e.g., RDF), while Web browsers request data from the resolver in a human-readable format (e.g., HTML). This solution is, in fact, a recommendation from the “Cool URIs” community of Web architects^17^. Such redirection-based resolvers involve a certain amount of duplication-of-effort since the codebase serving humans and the codebase serving machines are (sometimes) independent. Basic compliance with FAIR-ness, however, can be achieved in a more straightforward manner.

The lightweight solution we chose was to utilize XML Stylesheets to transform Linked Data, represented as RDF-XML for a machine, into the same data represented as HTML for a user's browser. Examples of other resources that have successfully adopted this approach include the transformations of the RDF representing SIO concepts^18^ and the transformations applied to the RDF data describing SADI Semantic Web Services^19^. The benefit of this approach is that there is only one interface, serving both humans and machines.

We implemented this it in the most simplistic way possible, thus fulfilling this challenging facet of FAIR-ness with minimal effort. The Perl code for our /Resource/ script, which acts as the URL resolver, is as follows:

#!/usr/local/bin/perl -w
use RDF::Query::Client;

my $URI = “http://.” $ENV{HTTP_HOST}. $ENV{REQUEST_URI};
my $query = RDF::Query::Client->new (’DESCRIBE <’.$URI.’>’);

my $iterator = $query->execute(’http://linkeddata.systems:8890/sparql’);
my $xml = $iterator->as_xml;

print “Content-Type: application/xml\n\n”;
**print ’<?xml version = “1.0” encoding=“UTF-8” ?>’.“\n”;**
**print ‘<?xml-stylesheet type=”text/xsl” href=“http://linkeddata.systems/styles/phi**.
**xsl” ?>′.“\n”;**

$xml =~ s/\<\?xml.*\>\n//;
print $xml;

The script first constructs the URI of the entity it is being asked to retrieve, using the path-information in the incoming HTTP request. This URI is then utilized in a “DESCRIBE” SPARQL query. The response to that query is a small Linked Data graph centered around the URI of interest. We then express that graph as RDF-XML, and make a small modification of the XML header (bold text) to include a reference to a transforming stylesheet, before passing the data back to the user's browser.

The stylesheet is similarly straightforward:

<xsl:stylesheet xmlns:phi=“http://linkeddata.systems/SemanticPHIBase/Resource/”
        xmlns=“http://www.w3.org/1999/xhtml”
        xmlns:xsl=“http://www.w3.org/1999/XSL/Transform”
        version=“2.0”>
<xsl:output method=“html” encoding=“utf-8” indent=“yes”/>
<xsl:variable name=“docroot” select=“//rdfs:label/../@rdf:about”/>
<xsl:variable name=“label” select=“//rdfs:label”/>
<xsl:template match=“/”>
<html>
<head><title><xsl:value-of select=“$label”/></title></head>
<body>
<h1>About: <xsl:value-of select=“$label”/></h1>
<**xsl:for-each select**=**“//rdf:Description[@rdf:about**=**$docroot]”>**
 <**xsl:for-each select**=“./*”>
   <**xsl:variable name**=**“url” select**=**“./@rdf:resource”**> <**/xsl:variable**>
   <**xsl:variable name="content" select**=**“.”**><**/xsl:variable>**
  <**h3**> <**xsl:value-of select**=**“name(.)”/**>**:  **<**/h3**>
  <**p style**=**“text-indent: 50px”**>
      <**xsl:choose**>
      <**xsl:when test**=**“$content** = **“”**>
     <**a href=’{$url}’**><**xsl:value-of select**=**“$url”/**><**/a**>
      <**/xsl:when**>
      <**xsl:when test**=**“not($content** = **”)”**>
     <**xsl:value-of select**=**“$content”/**>
     <**/xsl:when**>
     <**/xsl:choose**>
  <**/p**>
   <**/xsl:for-each**>
<**/xsl:for-each**>
</body></html>
</xsl:template>
</xsl:stylesheet>

The portion of the stylesheet in bold simply iterates over every triple in the RDF graph that that has the requested URI as the subject. Property values that are URLs are transformed into hyperlinks, and all other content is displayed as plain text.

Clearly, this does not result in an aesthetically rich rendering of the information for humans; however it is completely generic, and this transforming stylesheet could in principle be copy/pasted by any RDF provider, with minimal editing, to quickly fulfill this facet of FAIR-ness (“A”) for both humans and machines.

#### Repository-level FAIR-ness

We achieve the “R” facets of FAIR—pertaining to general domain-information, citation and license/usage metadata—by implementing software created by the FAIR Data “Skunkworks project”^20^ participants, called the FAIR Accessor. FAIR Accessors are compliant with the World Wide Web Consortium (W3C) Linked Data Platform (LDP^21^) design principles. The Accessor provides a top-level entry-point for machines to begin exploring the repository—the machine-equivalent of a “homepage.” The Accessor for PHI-base is:

http://linkeddata.systems/SemanticPHIBase/**Metadata**

Calling the URL of the Accessor invokes the Perl script called Metadata^22^ which responds by providing repository-level metadata, describing—through the use of ontologies and other domain vocabularies (i.e., compliant with FAIR “I”)—thecontent of the repository. It also includes authorship and other citation metadata, in both human-readable (names) and machine-readable (ORCID Identifier) formats, and human and machine-parsable license metadata. Importantly, in our implementation of the Accessor we attempted to also be compliant with both the content and vocabulary guidelines defined by the W3C Interest Group for Healthcare and Life Science Dataset Descriptions^23^ (Dumontier et al., 2016). The final list of repository-level metadata facets is shown in Table 1.

**Table 1:** **Metadata facets and their values in the repository metadata**.

**Vocabulary (prefix)**	**Metadata element**	**Semantic PHI-base value**
Dublin core (dc)	title	Semantic PHI-base
Resource Description Framework (rdf)	type	prov:Collection
Resource Description Framework (rdf)	type	dctypes:Dataset
Darpa Markup Language (daml)	has-Technical-Lead	“Dr. Alejandro Rodriguez Gonzalez,” “Alejandro Rodriguez Iglesias”
Darpa Markup Language (daml)	has-Principle-Investigator	“Dr. Mark Wilkinson,” “Dr. Kim Hammond-Kosack”
Provenance Authoring and Versioning (pav)	authoredBy	http://orcid.org/0000-0002-9699-485X
Provenance Authoring and Versioning (pav)	authoredBy	http://orcid.org/0000-0002-6019-7306
Friend of a Friend (foaf)	page	http://linkeddata.systems:8890/sparql
Friend of a Friend (foaf)	page	http://www.phi-base.org/
Dublin Core (dc)	creator	http://www.phi-base.org/
Dublin Core (dc)	issued	2015-11-17
Dublin Core (dc)	language	http://lexvo.org/id/iso639-3/eng
Data Catalogue Vocab. (dcat)	license	http://purl.org/NET/rdflicense/cc-by-nd4.0
Data Catalogue Vocab. (dcat)	identifier	http://linkeddata.systems/SemanticPHIBase/Metadata
Data Catalogue Vocab. (dcat)	keyword	“pathogenesis,” “plant/pathogen interactions,” “genetic database,” “phytopathology”
Data Catalogue Vocab. (dcat)	landingPage	http://www.phi-base.org/
Data Catalogue Vocab. (dcat)	description	FAIR Accessor server for the Semantic PHI-base. This server exposes the plant portion of the Pathogen Host Interaction database as Linked Data.
Data Catalogue Vocab. (dcat)	language	http://id.loc.gov/vocabulary/iso639-1/en
Data Catalogue Vocab. (dcat)	contactPoint	http://biordf.org/DataFairPort/MiscRDF/Wilkinson.rdf
Data Catalogue Vocab. (dcat)	publisher	http://wilkinsonlab.info, “Rothamsted Research,” http://www.rothamsted.ac.uk, http://www.phi-base.org
Data Catalogue Vocab. (dcat)	theme	http://linkeddata.systems/ConceptSchemes/semanticphi_concept_scheme.rdf

Several of these metadata facets are particularly noteworthy. The *dcat:theme* facet is intended to be the keyword list for machines. It is required by the DCAT ontology to have, as its value, a Simple Knowledge Organization System (SKOS) Concept Scheme^24^. Resolving the URL of semanticphi_concept_scheme.rdf returns an RDF document structured according to the requirements of SKOS, containing a list of domain-relevant external ontology concepts. Each of these concepts is identified by its URI, as well as its label and definition, thus again, being readable by both machines and people. The ontology terms we selected for Semantic PHI-base were, for example, “Bacterial Infectious Agent,” “Fungal Infectious Agent,” and “Plant Infectious Disease.” In addition, the page itself is formally typed as a “Collection” (from the PROV Ontology) and as a “Dataset” (from the Dublin Core Types vocabulary). These kinds of type-duplications are not harmful, but rather are created in an attempt to be comprehensible to as many automated agents as possible—some of which may know how to respond to only one or the other of these vocabularies. As stated in the FAIR Reusability principle, “To be Reusable metadata should have a plurality of accurate and relevant attributes,” implying that resource providers should strive for generosity in their provenance, rather than minimalism.

Finally, this first interaction with the FAIR Accessor also produces a (possibly paginated) list of all top-level items in the repository. In the case of PHI-base, these top-level items are the individual pathogen/host interactions. The format of this list is a series of additional newly-minted URLs, which represent meta-records—information *about* the individual records in Semantic PHI-base.

For Semantic PHI-base we chose to make these meta-record identifiers resolvable by the same Accessor code as provided the initial landing page. As such, the URLs of these meta-records have the structure similar to this example:

http://linkeddata.systems/SemanticPHIBase/**Metadata**/INT_00001

where /Metadata is the same script that served as the primary entry-point of the Accessor. The Accessor script receives this RESTful URL, and extracts the identifier /INT_00001 from the URLs path. It uses this information to execute a record-specific SPARQL query against Semantic PHI-base. It then creates a series of RDF statements containing record-specific information, and adds this to other preconfigured general metadata. These together act as the record-level metadata. Finally, two additional links are generated, utilizing the DCAT ontology vocabulary and structure, that point to the original record in PHI-base, explicitly typed as being HTML (for humans), and a link to the same record in Semantic PHI-base, explicitly typed as being RDF (for machines). This package of information is returned to the user or their computational agent, who then decides which of those two URLs is appropriate to follow in order to retrieve the PHI-base data record.

An example of this record-level metadata is shown below, with the bolded sections showing the trail leading from the meta-record, through to the retrieval instructions for the machine-readable and human-readable records.

@prefix dc11: <http://purl.org/dc/elements/1.1/>.
@prefix ns0: <http://purl.org/pav/>.
@prefix void: <http://rdfs.org/ns/void#>.
@prefix ns1: <http://www.ksl.stanford.edu/projects/DAML/ksl-daml-desc.daml#>.
@prefix dcat: <http://www.w3.org/ns/dcat#>.
@prefix foaf: <http://xmlns.com/foaf/0.1/>.

<http://linkeddata.systems/SemanticPHIBase/Metadata/INT_00004>
dc11:creator <http://www.phi-base.org/>;
dc11:issued “2015-11-17”;
dc11:license <http://purl.org/NET/rdflicense/cc-by-nd4.0>;
dc11:title “PHI-Base Interaction PHI:PHI:11”;
ns0:authoredBy <http://orcid.org/0000-0002-6019-7306>, <http://orcid.org/0000-0002-9699-485X>;
void:inDataset <http://linkeddata.systems/SemanticPHIBase/Metadata>;
ns1:has-Principle-Investigator “Dr. Kim Hammond-Kosack”, “Dr. Mark Wilkinson”;
ns1:has-Technical-Lead “Alejandro Rodriguez Iglesias”, “Dr. Alejandro Rodriguez Gonzalez”;
dcat:contactPoint <http://biordf.org/DataFairPort/MiscRDF/Wilkinson.rdf>;
dcat:description “RDF representation of PHI Base Interaction Record PHI:PHI:11”;
**dcat:distribution**  <**http://linkeddata.systems/SemanticPHIBase/Resource/interaction/INT_00004**>,
         <**http://www.phi-base.org/query.php?detail=yes&phi_acc=PHI:11>;**
dcat:identifier <http://linkeddata.systems/SemanticPHIBase/Metadata>;
dcat:keyword “PHI Base”, “host/pathogen interaction”, “pathogenesis”;
dcat:landingPage <http://www.phi-base.org/>;
dcat:language <http://id.loc.gov/vocabulary/iso639-1/en>;
dcat:license <http://purl.org/NET/rdflicense/cc-by-nd4.0>;
dcat:modified “2015-11-17”;
dcat:publisher <http://wilkinsonlab.info>, <http://www.phi-base.org>, <http://www.rothamsted.ac.uk>, “Rothamsted Research”;
foaf:page <http://www.phi-base.org/>.

<http://linkeddata.systems/SemanticPHIBase/Resource/interaction/INT_00004>
**dc11:format “application/rdf+xml”;**
**a** <**http://purl.org/dc/dcmitype/Dataset**>**, void:Dataset, dcat:Distribution;** **dcat:downloadURL**<**http://linkeddata.systems/SemanticPHIBase/Resource/interaction/INT_00004****>**.

<http://www.phi-base.org/query.php?detail=yes&phi_acc=PHI:11>
**dc11:format “text/html”;**
**a** <**http://purl.org/dc/dcmitype/Dataset**>**, dcat:Distribution;**
**dcat:downloadURL**<**http://www.phi-base.org/query.php?detail=yes&phi_acc=PHI:11**>.

While this behavior is, arguably, somewhat complex, the FAIR Data project has provided downloadable libraries in Perl that manage most of these behaviors. The required modules can be installed from CPAN (FAIR::Data), or from the FAIR Data Initiative GitHub^25^. Using this codebase, implementation of the Accessor layer primarily involves setting key/value pairs, where most of the keys are pre-defined. In the case of Semantic PHI-base, our implementation of the Accessor required less than one person-day, with most of the effort directed to the identification of descriptive ontology terms for the SKOS model (the value of the *dcat:theme* metadata facet).

#### Connecting data publication to metadata publication

While all FAIR principles have already been addressed, there is one additional FAIR desiderata that would make the data itself more compliant with the spirit and intent of the principles. SPARQL queries over Semantic PHI-base provide the user with appropriate responses; however this data lacks provenance information. It would be more FAIR (particularly with respect to “R”—Reusability) if there were a mechanism for retrieving provenance data from the SPARQL query itself. One simple, but possibly non-obvious solution is to use the URL of the FAIR Accessor as the identifier of the named graph containing the PHI-base RDF within the triplestore. This, therefore, explicitly connects all data in Semantic PHI-base with the identifier of the repository, which itself resolves to comprehensive metadata about the repository which is suitable for journal citation or other citation purposes. Examples of such queries are provided in the Results and Discussion section.

## Results and discussion

While the Methods section was structured according to a data publication workflow, and therefore addressed the FAIR Data Principles in a somewhat piecemeal manner, this discussion will organize itself around the four FAIR Principles, explaining in-turn how each of them are addressed. This will highlight how the overall transformation, and the decisions made at each step, addresses each of the principles.

### Meeting the fair principles

#### Principle F: findable

The principle of Findability focuses on the unique and unambiguous identification of all relevant entities; the rich annotation and description of these entities; the searchability of those descriptive annotations; and the explicit connection between metadata and data elements. Semantic PHI-base addresses each of those points as follows.

- *Identified*: The Repository Accessor provides a globally unique identifier for the Semantic PHI-base resource overall—its URL. This URL provides access to meta-identifiers for each record within the repository. Going deeper, our core data model, based on SIO, requires us to create identifiable entities for every data element, including numerical and text fields. As such, every element of Semantic PHI-base is explicitly, unambiguously, and uniquely identified, from the repository level all the way down to the individual data values within each record. In the case of locally-defined data elements, the identifiers are a locally-minted URI. In the case of shared concepts, the community-preferred identifier is selected.
- *Annotated*: Where possible—that is, where such information is available from PHI-base—identified entities carry their provenance metadata. For example, *Interaction Context* entries carry with them any available *Protocol* information, describing how that data came-to-be. While there is little high-granularity metadata within PHI-base, our selection of the SIO core model ensures that there is a place to include such metadata, should it become available in the future.
- *Searchable*: The Repository Accessor URL, and the Accessor's URLs for each meta-record, are publicly available by HTTP GET, making all of this repository and record metadata accessible to search engines such as Google. Publication of the data triples in a publicly available SPARQL endpoint allows both humans and machines to search for data of interest, and discover the unique identifier for any record or sub-element within that record.
- *Connected*: There is a symmetrical link between data and metadata in Semantic PHI-base. The metadata provided by the Accessor includes the identifiers of individual records, along with their formats. Conversely, the named-graph that contains the records within the SPARQL endpoint utilizes the URI of the Accessor, allowing both humans and machines to traverse from metadata, to data, or vice versa, using fully standards-compliant protocols.

#### Principle A: accessible

The principle of Accessibility speaks to the ability to retrieve data or metadata based on its identifier, using an open, free, and universally implementable standardized protocol. The protocol must support authentication and authorization if necessary, and the metadata should be accessible “indefinitely,” and independently of the data, such that identifiers can be interpreted/understood even if the data they identify no longer exists. We address this principle in the following ways:

- *Accessibility*: Semantic PHI-base accomplishes most features of FAIR Accessibility by using URLs to identify all entities. The provision of a Resolver for all locally-coined URLs ensures that all (meta)data can be accessed using the open global standard HTTP protocol, including HTTP-level authentication if necessary.
- *Longevity and Independence*: We achieve data/metadata independence by explicitly creating two additional “layers” above the core dataset. The Accessor provides both repository- and record-level metadata, using identifiers that are distinct from the identifiers of the data elements themselves (though, as noted above, there is a symmetrical cross-reference between these). As such, the metadata for the repository, and the metadata for each core record, will (can) continue to exist even if the SPARQL endpoint containing the data disappears.

#### Principle I: interoperable

The Interoperability Principle states that (meta)data use a formal, accessible, shared, and broadly applicable language for knowledge representation; that vocabularies themselves should follow FAIR principles; and that the (meta)data should include qualified references to other (meta)data. Semantic PHI-base accomplishes these goals in the following ways:

- *Knowledge Representation Language*: Semantic PHI-base uses two globally-accepted standards for knowledge representation—syntactic and semantic—in the form of RDF and OWL, respectively. These are non-domain-specific data and knowledge representation technologies, that are therefore highly-applicable to our objective of interoperability and integration.
- *FAIR Vocabularies*: We address this aspect of FAIR-ness in two ways. First, we make an extensive effort to map all PHI-base concepts into vocabularies/ontologies created by others. We limit ourselves to those ontologies that are published openly, and for which the vocabulary's concept identifiers resolve according to FAIR Principles (for example, OBO ontologies and ontologies from the W3C). Second, when a concept does not map into any third-party vocabulary, we assign it a novel and globally-unique identifier, and we publish that concept in a location that can be accessed based on that identifier, using the standard HTTP protocol.
- *Qualified Cross-Referencing*: This is achieved largely by our use of RDF, and our emphasis on providing rich metadata at all levels—from the repository-level down to sub-data-element level. RDF, by design, must specify the purpose or meaning of the link between any two data entities, using a globally unique concept identifier to express that purpose/meaning. We further ensure that all of the links utilized by Semantic PHI-base resolve to a description of that meaning, using the HTTP protocol, to ensure both human and machine-readability. We avoid overly-general, ambiguous cross-reference qualifiers such as “see also,” preferring more specific descriptive link-descriptors whenever possible. Finally, we create a thin software layer that generates a human-readable, Web-browser-friendly display of this RDF data, to support human users' exploration via exactly the same interface as is used by the machine, thus reducing redundancy and code-duplication.

#### Principle R: reusable

The FAIR Reusability principle requires that meta(data) have a plurality of accurate and relevant attributes; provide a clear and accessible data usage license; associate data and metadata with their provenance; and meet domain-relevant community standards for data content.

- *A plurality of attributes*: Every data element in Semantic PHI-base is explicitly typed according to its category in our semantic model. Where appropriate, however, we go further, and provide multiple possible interpretations of the data element's type, using a multiplicity of third-party categorization systems and ontologies. In doing so, we broaden the scope of (in particular) machine-accessibility by mapping PHI-base concepts into multiple, publicly-accepted knowledge models, each of which may only be understood by a subset of computational data-harvesting agents.
- *License and Provenance*: At the repository level, we attempt to provide metadata spanning attributes including license, authorship, citation, contact information, domain and scope of the repository, and temporal/curatorial information. This metadata is associated with the data via the named-graph/Accessor URL. Moreover, we emphasize both the human and machine-readable requirements of FAIR by, for example, utilizing both an author's name, and their corresponding ORCID identifier in the form of its HTTP-resolvable URL. Similarly, with respect to the scope of the repository, we provide third-party ontological descriptors in both machine and human-readable form.
- *Community-relevant standards*: While there are no community-accepted standards for Pathogen/Host interaction data (such as the “minimal information” standards found in other data domains such as microarrays or proteomics), we nevertheless utilize community-accepted standards at the dataset descriptor level. For example, we adhere to the W3C Interest Group for Healthcare and Life Science Dataset Descriptions standard in the repository-level metadata.

As demonstrated by the detailed discussion above, Semantic PHI-base addresses, and in many cases exceeds, all requirements of FAIR-ness. Notably, it addresses these requirements paying close attention to supporting both human and computationally-driven data exploration.

### Beyond being fair—added value that results from fair data publishing

The FAIR Principles lay-out the foundational expectations for contemporary scholarly data publishing; adherence to these principles is, in itself, an important step toward transparent, reusable data, and will increasingly become something that is expected of researchers. This section addresses what other benefits are gained by becoming a FAIR data publisher, beyond simply meeting the expectations of the scholarly community and the agencies and governments that support them.

#### Integrative queries using a global standard

Given the attention paid to data harmonization, and to the linkage and interoperability aspects of FAIR-ness, it is now possible to ask questions from this FAIR dataset that are not possible to ask from the original PHI-base. All of the following queries can be copy/pasted into the SPARQL query box provided by the project^26^ and the data resides in its own named graph^27^. For all queries, the prefixes are:

PREFIX RO: <http://purl.obolibrary.org/obo/ro.owl#>
PREFIX SIO: <http://semanticscience.org/resource/>
PREFIX EDAM: <http://edamontology.org/>
PREFIX PHIO: <http://linkeddata.systems/SemanticPHIBase/Resource/>
PREFIX PUBMED: <http://linkedlifedata.com/resource/pubmed/>
PREFIX rdfs: <http://www.w3.org/2000/01/rdf-schema#>
PREFIX up: <http://purl.uniprot.org/core/>
PREFIX foaf: <http://xmlns.com/foaf/0.1/>
PREFIX skos: <http://www.w3.org/2004/02/skos/core#>
PREFIX schema: <http://schema.org/>

With increasing complexity, we now demonstrate how to explore the content of Semantic PHI-base, and further, how to integrate it with data from other databases on the Web.

**Query 1: Retrieve an overview of the interactions in Semantic PHI-base**.

SELECT DISTINCT ?s ?l
WHERE {
   ?s a PHIO:PHIBO_00022.
   ?s rdfs:label ?l
}

**Query 2: What organisms are hosts for *Magnaporthe* species?**

SELECT DISTINCT(str(?species2)) as ?hostspecies

WHERE {
   ?int a PHIO:PHIBO_00022.
   ?int PHIO:has_participant ?part1.
   ?int PHIO:has_participant ?part2.
   FILTER(?part1 != ?part2).

   ?part1 PHIO:is_member_of ?organism1.
   ?organism1 rdfs:label ?species1.
   FILTER (CONTAINS (?species1, “Magnaporthe”)).

   ?part2 PHIO:is_member_of ?organism2.
   ?organism2 rdfs:label ?species2.
}

**Query 3: What genes affect *Magnaporthe* infection, and what are the phenotypic outcomes from allele variants, organized by host species?**

SELECT DISTINCT ?int str(?species1) as ?Pathogen str(?genename) as ?Locus
str(?species2) as ?Host str(?pheno) as ?Phenotype

WHERE {
   ?int a PHIO:PHIBO_00022.

   ?int PHIO:has_participant ?part1.
   ?int PHIO:has_participant ?part2.
   FILTER(?part1 != ?part2).

   ?part1 PHIO:is_member_of ?organism1.
   ?organism1 rdfs:label ?species1.
   FILTER(CONTAINS(?species1, “Magnaporthe”)).

   ?part2 PHIO:is_member_of ?organism2.
   ?organism2 rdfs:label ?species2.

   ?gene PHIO:is_proper_part_of ?organism1.
   ?gene PHIO:has_unique_identifier ?geneid.
   ?geneid a EDAM:data_2299.
   ?geneid PHIO:has_value ?genename.

   ?int PHIO:is_manifested_as ?intcont.
   ?intcont PHIO:has_quality ?desc.
   ?desc PHIO:has_value ?pheno.
   FILTER(!str(?pheno) = “BASE STATE”)

}
ORDER BY ?organism2

An advantage of the semantic representation, and a key reason that we utilized the Identifiers.org URI scheme in our RDF representation, was to facilitate dynamic federation with third-party datasets on the Web. Some examples of federated queries, utilizing the Identifiers.org cross-reference lookup service, are:

**Query 4: Retrieve images of *Magnaporthe* host species from UniProt**.

SELECT ?hostspecies ?picture

WHERE {
   {
   SELECT DISTINCT(str(?species2) as ?hostspecies) ?organism2
     WHERE {
        ?int a PHIO:PHIBO_00022.

        ?int PHIO:has_participant ?part1.
        ?int PHIO:has_participant ?part2.
        FILTER(?part1 != ?part2).

        ?part1 PHIO:is_member_of ?organism1.
        ?organism1 rdfs:label ?species1.
        FILTER(CONTAINS(?species1, “Magnaporthe”)).

        ?part2 PHIO:is_member_of ?organism2.
        ?organism2 rdfs:label ?species2.
        FILTER(CONTAINS(str(?organism2), “identifiers.org”))
      }
     }

   SERVICE <http://identifiers.org/services/sparql> {
        ?organism2 owl:sameAs ?crossref.
        FILTER(CONTAINS(str(?crossref), “purl.uniprot”))
   }

   SERVICE <http://sparql.uniprot.org/sparql> {
        OPTIONAL{?crossref foaf:depiction ?picture}
   }
}

**Query 5: What are the Gene Ontology annotations of the *Magnaporthe* genes with mutant alleles that cause hypervirulence (increased disease causing ability) on the host plant rice?**

SELECT DISTINCT (str(?species1) as ?Pathogen) (str(?genename) as ?Locus) ?protid
?uniprot (str(?species2) as ?Host) (str(?pheno) as ?Phenotype) ?goterm

WHERE {
    {
   SELECT DISTINCT ?protid ?species1 ?genename ?species2 ?pheno
   WHERE {
        ?int a PHIO:PHIBO_00022.

        ?int PHIO:has_participant ?part1.
        ?int PHIO:has_participant ?part2.
        FILTER(?part1 != ?part2).

        ?part1 PHIO:is_member_of ?organism1.
        ?organism1 rdfs:label ?species1.
        FILTER(CONTAINS(?species1, “Magnaporthe”)).

        ?part2 PHIO:is_member_of ?organism2.
        ?organism2 rdfs:label ?species2.
        FILTER(CONTAINS(?species2, “Rice”)).

        ?gene PHIO:is_proper_part_of ?organism1.
        ?gene PHIO:has_unique_identifier ?geneid.
        ?geneid a EDAM:data_2299. # Gene Name
        ?geneid PHIO:has_value ?genename.

        ?gene PHIO:has_unique_identifier ?protid.
        ?protid a EDAM:data_2291. # UniProt Identifier

        ?int PHIO:is_manifested_as ?intcont.
        ?intcont PHIO:has_quality ?desc.
        ?desc PHIO:has_value ?pheno.

        FILTER(CONTAINS(str(?pheno), “Increased virulence”))
      }
   }

   SERVICE <http://identifiers.org/services/sparql> {
     ?protid owl:sameAs ?uniprot.
     FILTER(CONTAINS(str(?uniprot), “purl.uniprot”))
   }

   SERVICE <http://sparql.uniprot.org/sparql> {
        ?uniprot up:classifiedWith ?annot.
        ?annot rdfs:label ?goterm.
        ?annot a owl:Class.
   }
}

**Query 6: What are the MeSH terms associated with publications describing interactions between *Magnaporthe* and wheat?**

select distinct ?intlabel ?pubmed ?meshterm
where {
   {select distinct ?pubmed ?intlabel
    where {
        {select distinct ?pmid ?intlabel
         where {
           ?int a PHIO:PHIBO_00022.
           ?int rdfs:label ?intlabel.
           ?int PHIO:has_participant ?part1.
           ?int PHIO:has_participant ?part2.
           FILTER(?part1 != ?part2).

           ?int PHIO:is_manifested_as ?intcont.
           ?intcont PHIO:is_output_of ?investigation.
           ?investigation schema:citation ?cite.
           ?cite PHIO:has_unique_identifier ?pmid.

           ?part1 PHIO:is_member_of ?organism1.
           ?organism1 rdfs:label ?species1.
           ?part2 PHIO:is_member_of ?organism2.
           ?organism2 rdfs:label ?species2.

           FILTER(CONTAINS(?species1, “Magnaporthe”)).
           FILTER(CONTAINS(?species2, “Wheat”)).
         }}

         SERVICE <http://identifiers.org/services/sparql> {
           ?pmid owl:sameAs ?pubmed.
           FILTER(CONTAINS(str(?pubmed), “linkedlifedata”))
          }

   }}

SERVICE <http://linkedlifedata.com/sparql/> {
  OPTIONAL {?pubmed PUBMED:meshHeading ?mh.
  ?mh PUBMED:mesh ?mesh.
  ?mesh rdfs:label ?meshterm}
   }
}

**Finally, the location of provenance information for Semantic PHI-base can be retrieved by requesting the URL of the named graph. For example in Query 7:**

SELECT DISTINCT **?provenance** ?s ?l
WHERE {
   GRAPH **?provenance** {
   ?s a PHIO:PHIBO_00022.
   ?s rdfs:label ?l
   }
}

Visiting the URL that is assigned to the ?provenance variable will retrieve the metadata shown in Table 1. As such, we have fulfilled the requirement of FAIR that full provenance information is provided with every data-point.

These queries demonstrate that the utility of the provider's data is enhanced by a FAIR representation. More importantly, it becomes easier to provide a richer data exploration experience for the resource's target users.

#### Transparent, yet fine-grained access control

Being FAIR, however, is more than merely converting the data into RDF format to support queries. In fact, the FAIR Principles do not require the data to be available for query at all. The FAIR Principles perspective on data openness speaks only to transparency in licensing and access procedures, making no statements whatsoever regarding the openness of access to the data itself. Therefore, it is useful to point out the significant benefits that arise from generating and publishing rich metadata, since this additional effort is a requirement for FAIR data publishing.

One example of a benefit is that it becomes possible to provide informative, yet fine-grained access control over the data. Although issues of privacy were not addressed by this work, our FAIR transformation methodology has features that aim to support data publishers with privacy concerns, or who hold data with restricted access such as commercial or pay-for-use data. In such cases, it is the FAIR metadata that supports findability and reusability. For Semantic PHI Base, a small piece of software—the FAIR Accessor—provided rich metadata describing the general content of the resource such as the types of pathologies within the records, the range of organisms, etc. These metadata would enable a user, or their agent, to decide if the database is of-interest to them, given their current task. Moreover, it contains explicit license information allowing that user/agent to determine their right to access the data, and the process for requesting access if they do not have it. At the provider's discretion, and depending on the sensitivity of the data, they may also provide the second layer of the FAIR Accessor, which issues record-level metadata which may provide additional information about the nature of the content of that specific record.

The FAIR Principles hold that you should not presume a specific end-use for your data, but rather provide sufficient metadata to support a wide range of potentially unexpected end-uses. The metadata produced by the two levels of the FAIR Accessor could be used by, for example, project administrators, funding agencies, or third-party catalogues/registries, who wish to know how many records exist in total, how many records relate to a specific condition or trait, or other high-level demographic information. Thus, open provision of FAIR metadata, even in the absence of open data publication or FAIR transformaton, has significant advantages for a wide range of stakeholders, while giving the provider significant control over what is made public. The value of this added effort will only increase as tools become available that can effectively crawl, interpret, and index FAIR metadata.

#### The benefit of FAIR for users and peer data publishers

Data users and other data publishers parties may benefit from this FAIR representation in three core ways.

First, the FAIR representation and publication of PHI Base data enhances the ability for third-parties to synchronize their data holdings with the regularly curated, domain-specific data within PHI-base. Moreover this synchronization can be achieved without the need for a bespoke query and data extraction tool, as would be required to interrogate the current Web interface or XML files provided by PHI Base. In the future we anticipate that, for example, the Gene Ontology may use Semantic PHI-base to dynamically retrieve new protein annotations curated by the PHI-base expert community of biologists, or UniProt may use it to retrieve Pathogen-Host protein interaction data that have experimentally proven pathogenicity.

Second, there is a predictable way of moving from a record, or even a data component within that record, to relevant metadata that describes: what that data is about; if there are other similar data records available in the resource; how/if they may be used, by whom, for what purpose; and how it should be cited. On the Web at present, if this can be done at all, it must be done by visiting the website of the data resource, manually exploring that website to find the license and usage-terms, and exploring it again to find provider-suggested citation information (if such exists). Understanding the broader context and content of the entire resource would generally require reading the cited manuscript. Through the FAIR data publishing approach, this is addressed by creating symmetrical linkages from repository-level references all the way down to individual records and even their sub-components. This results in a predictably-structured, machine-traversable path both from top-down, and from bottom-up, ensuring that Semantic PHI Base data can always be contextualized, even if the URI for a PHI Base data element is encountered in isolation from the database itself (for example, in a third-party database, a blog, a tweet, or an email attachment).

Third, all data elements and sub-elements are named, resolvable, and reusable. This ensures that, not only can data elements always be contextualized, as mentioned above, but that all data elements can be “bookmarked,” and/or referred-to by others. Thus, third parties can add their own information to any record or record-component within Semantic PHI Base, without ambiguity, by making statements about the relevant URI; humans or automated agents exploring that third party data may then look-up these references in Semantic PHI Base in a predictable way.

### Additional observations, considerations, and limitations

While designing the Semantic PHI-base model, we specifically looked toward future integration and cross-query of the PHI-base data with two specific external information sources—the data in the Plant Pathogen Interaction Ontology and knowledgebase (Iglesias et al., 2013), and the Ortholog Ontology (Fernández-Breis et al., 2015). The former is also modeled on the disease triangle, and contains more details about the mechanisms of pathogenicity which could be used to construct mechanism-oriented queries via federation. The latter contains large-scale gene orthology information based on taxonomy, thus our inclusion of taxonomy nodes in the PHI-base semantic model will allow us to begin cross-species queries based on the PHI-base genetic data.

This enhanced power justifies the effort of not adopting a trivial approach to RDF conversion. For example, it would have been straightforward to assign every XML tag in the PHI Base data dump to a novel RDF predicate, then capture the value of that field as a literal value of that property. This, however, would have resulted in a data structure that did not meet many of the FAIR objectives—in particular, the objective of machine-readability and automated integration.

Similarly, the extensive use of ontologies, as required by the FAIR Principles, has notable benefits particularly for mechanized access. For example, on the Web, sequence cross-references such as EMBL, UniProt, and RefSeq ID numbers are generally published as strings of characters that are (usually) hyperlinked. Human consumers will look at the cross-reference in their Web browser and will know—either from the layout and/or labels on the Web page, or by experience based on recognizing the pattern of letters and numbers—which entries correspond to which database. This is generally not true for machines. When providing machine-readable data, it is necessary to make all contextual information explicit and transparent. In the Semantic PHI-base RDF, not only do we represent these sequence cross-references using the canonical URL from Identifiers.org, in place of a simple ID number, but we also use the EDAM ontology to explicitly indicate what type of identifier that is. For example:

  http://identifiers.org/uniprot/P22287
rdf:type http://edamontology.org/data_3021

where EDAM:data_3021 is the concept “UniProt Accession.” Thus, there is no ambiguity, from the perspective of an automated agent, regarding what kind of identifier is being provided; an agent that has been tasked to retrieve protein entries corresponding to a certain subset of records, will be able to accomplish this task without manual intervention. Therefore, by attempting to adhere to the *spirit* of the FAIR data principles—as opposed to simply “RDF-izing” the data—the result will be easier for both machines and humans to discover, reuse and cite, thus enhancing the utility, and the scholarly impact, of both the repository and the data it contains.

The FAIR publishing approach is emergent, and therefore has some limitations that often prevent it from being fully exploited. Federated query, for example, requires that the third-party data also have a semantic representation, and that it is available on a public SPARQL endpoint, or as a downloadable RDF file. There are still relatively few bioinformatics databases or annotation resources that have Linked Data representations (though the critical data providers such as UniProt and EBI are increasingly publishing their contents in RDF format). In the case of some other important resources, their content has been transformed and warehoused by third parties (such as the PubMed RDF provided by Linked Life Data and Bio2RDF). These warehouses, however, have a variety of weaknesses: they are prone to being out-of-date, since the conversion of the primary resource into RDF is done on an (often arbitrary) schedule; they are often hosted by providers with limited resources, and thus may suffer significant downtime or low quality-of-service; and finally, they are non-canonical, thus the consumer must trust the provider to have properly converted the resource, and to have converted the portions that are of-interest to the user. As discussed in the Introduction, we believe that the increasing pressure from funding agencies and publishers to improve the FAIR-ness of scholarly data will soon remedy this problem, and that increasingly, core bioinformatics resources will be natively published in formats that are findable, accessible, interoperable, and reusable.

## Conclusion

Achieving a FAIR data ecosystem within the plant sciences allows us to better-serve our community of researchers, enabling access for both humans and machines, and thereby facilitating the large-scale exploration, analysis, and reuse of these valuable resources. We describe one possible approach to achieving comprehensive FAIR-ness, and demonstrate that many FAIR principles are, in practice, straightforward to implement using off-the-shelf technologies and standards. All tools and code for this project are available in the project GitHub^28^. We hope that the outcomes reported here will inspire the thousands of other life science data resources to begin similar transformations, and that these guideposts to implementation of the FAIR Data Principles will facilitate them in this process.

## Author contributions

ARI and ARG contributed equally to this work by designing the FAIR data model, implementing the conversion, generating the mapping files, and evaluating the results. AGI, MU, and KHK collected, organized, and published the original data, provided advice on the semantics of their data model, edited the manuscript, and tested the validity of queries. AS and MW co-supervised all activities of AI, read and revised the manuscript. MW wrote the manuscript and supervised all facets of the project.

## Funding

AS was funded by the Spanish Research council (MICINN, grant ref. BIO2014-53211-R), the EC (REA grant agreement n. 304039) and the Community of Madrid (grant ref. S2013/ABI-2734).

### Conflict of interest statement

The authors declare that the research was conducted in the absence of any commercial or financial relationships that could be construed as a potential conflict of interest.

## Footnotes

^1^http://figshare.com

^2^http://www.datadryad.org/

^3^https://data.mendeley.com/

^4^http://zenodo.org/

^5^http://datahub.io

^6^http://www.dans.knaw.nl

^7^http://ec.europa.eu/research/participants/data/ref/h2020/grants_manual/hi/oa_pilot/h2020-hi-oa-data-mgt_en.pdf

^8^http://datafairport.org/fair-principles-living-document-menu

^9^https://sites.google.com/site/rdbyod/home

^10^http://www.PHI-base.org

^11^http://www.phi-base.org/help.php#curation

^12^http://obofoundry.org/id-policy.html

^13^http://purl.org

^14^https://www.blazegraph.com/product/

^15^https://jira.blazegraph.com/browse/BLZG-1484

^16^https://github.com/openlink/virtuoso-opensource/issues/244

^17^http://www.w3.org/TR/cooluris/

^18^http://semanticscience.org/resource/SIO_000008.rdf

^19^http://biordf.org/cgi-bin/SADI/OpenLifeData2SADI/SADI/omim/omim_vocabulary_Gene-phenotype_omim_vocabulary_x-flybase (note that this URL will not be converted to HTML by the Chrome browser)

^20^http://www.slideshare.net/markmoby/force11-jddcp-workshop-presentation-43421243

^21^http://www.w3.org/TR/ldp/

^22^https://github.com/wilkinsonlab/SemanticPHIbase/blob/master/Interface/Perl/Metadata

^23^http://www.w3.org/TR/hcls-dataset/

^24^http://www.w3.org/2009/08/skos-reference/skos.html

^25^https://github.com/FAIRDataInitiative/DataFairPort/tree/master/Libraries/Perl/FAIR

^26^http://sparql.linkeddata.systems/sparql

^27^http://linkeddata.systems/SemanticPHIBase/Metadata

^28^http://github.com/wilkinsonlab/SemanticPHIbase
